# Efficacy and safety of carbon ion radiotherapy for chordomas: a systematic review and meta-analysis

**DOI:** 10.1186/s13014-023-02337-x

**Published:** 2023-09-13

**Authors:** Meng Dong, Ruifeng Liu, Qiuning Zhang, Dandan Wang, Hongtao Luo, Yuhang Wang, Junru Chen, Yuhong Ou, Xiaohu Wang

**Affiliations:** 1 The First School of Clinical Medicine, Lanzhou University, Lanzhou, China; 2 Institute of Modern Physics, Chinese Academy of Sciences, No.1, Yanxia Road, Chenguan District, Lanzhou, 730030 People’s Republic of China; 3 Department of Postgraduate, University of Chinese Academy of Sciences, Beijing, China; 4 Heavy Ion Therapy Center, Lanzhou Heavy Ions Hospital, Lanzhou, China

**Keywords:** Carbon ion, Radiotherapy, Charged particle, Chordoma, Meta-analysis

## Abstract

**Objective:**

Carbon ion radiotherapy (C-ion RT) for chordomas has been gradually performed in several research centres. This study aimed to systematically review the results of clinical reports from these institutions and to evaluate the safety and efficacy of C-ion RT.

**Methods:**

In accordance with the PRISMA guidelines and set search strategies, we searched four databases for articles from their inception to February 11, 2023. These articles were screened, and data were extracted independently by two researchers. STATA 14.0 was used for statistical analysis of survival results.

**Results:**

A total of 942 related articles were retrieved, 11 of which were included. Regarding lesion location, 57% (n = 552) originated in the sacral region, 41% (n = 398) in the skull base, and 2% (n = 19) in the spine (upper cervical). The local control (LC) rates at 1, 2, 3, 5, 9, and 10 years in these studies were 96%, 93%, 83%, 76%, 71%, and 54%, respectively. The overall survival (OS) rates at 1, 2, 3, 5, 9, and 10 years in these studies were 99%, 100%, 93%, 85%, 76%, and 69%, respectively. Acute and late toxicities were acceptable, acute toxicities were mainly grade 1 to grade 2 and late toxicities were mainly grade 1 to grade 3.

**Conclusion:**

C-ion RT has attractive clinical application prospects and is an important local treatment strategy for chordomas. Encouraging results were observed in terms of LC and OS. Meanwhile, the acute and late toxicities were acceptable.

*PROSPERO registration number*: CRD42023398792.

**Supplementary Information:**

The online version contains supplementary material available at 10.1186/s13014-023-02337-x.

## Introduction

Chordomas are locally aggressive malignant bone tumours with a low incidence that arise from cells of notochord remnants. Thus, chordomas are often located in the midline from the skull base to the sacrococcygeal bone [1]. Overall, chordomas have an incidence of 0.8–1 per million, with approximately 50–55% of cases located in the sacrococcygeal bone, followed by the clival region (30–35%) and mobile spine (10–20%) [2–6]. Chordomas have a low potential for metastasis, and the preferred treatment strategy for them is complete surgical resection [7]. Previous studies have reported that local control (LC) is a significant survival outcome [7]. However, complete resection of some chordomas are often difficult in patients with adjacent to critical anatomical structures such as the brainstem, spinal cord, and optic pathways [8, 9]. Radiotherapy (RT) plays an irreplaceable role in the treatment of chordomas, especially those that are inoperable or have residual tumours after surgery [10].

Chordomas are known to have low radiosensitivity, requiring higher-dose irradiation (at least 70 Gy) to gain adequate LC [11]. However, because of the proximity of some chordomas to critical risk organs, such as the brain stem, spinal cord, and optic nerve pathways, achieving high doses of tumour irradiation can be difficult with traditional photon therapy. In recent decades, particle radiotherapies, such as proton beam therapy (PBT) and carbon ion radiotherapy (C-ion RT), have emerged as RT techniques. A higher dose can be delivered to the tumour area (Bragg peak effect) compared to photons while protecting the organs at risk (OARs). Compared with PBT and photons, C-ion RT has better relative biological effectiveness (RBE). Considering the abovementioned technical advantages, C-ion RT can become a new treatment option for chordomas [12].

In recent years, C-ion RT for chordomas has been gradually performed in several research centres. This study aimed to systematically review the results of clinical reports from these institutions and to evaluate its safety and efficacy.

## Materials and methods

### Literature identification

This study was registered in the International Prospective Register of Systematic reviews (PROSPERO) (Registration No. CRD42023398792). This systematic review and meta-analysis adhered to the PRISMA guidelines and recommendations [13].

### Search strategy

Candidate articles were obtained by searching four databases (Cochrane Library, Embase, PubMed, and Web of Science) from their inception to 11 February 2023. Literature not written in English was excluded. The search terms were as follows: ((“Chordoma OR Chordomas OR Chordoma*”) AND (“Heavy Ion Radiotherapy OR Heavy Ion Radiotherapies OR Heavy Ion Therap* OR Heavy Ion Radiation Therapy OR Carbon Ion Radiotherapy OR Carbon Ion Therap* OR Carbon Ion Radiation Therapy OR C-ion therapy OR hadron OR particle OR charged particle”)). In addition, we traced the relevant references and manually searched the abstracts of congress meetings.

### Inclusion and exclusion criteria

These articles were screened, and data were extracted independently by two researchers (MD and QZ). The inclusion criteria were as follows: (a) all patients were pathologically diagnosed with chordoma; (b) patients received C-ion RT; and (c) reported incidence of toxicity and survival outcomes, including overall survival (OS) and LC from initial diagnosis. The exclusion criteria were as follows: (a) studies on patients receiving other RT techniques, including photons, PBT, brachytherapy, and other charged particles; (b) duplicate publications; (c) overlapping cohorts (only the most complete studies were included); (d) re-irradiation studies; (e) sample size of < 10 patients; (f) lack of detailed data; and (g) other irrelevant studies (case reports, reviews, and protocols).

### Data extraction

Data extraction was performed independently by two reviewers (RL and QZ), and the results were verified by a third reviewer (DW). The following data were extracted: (a) research institution, study period, and study design; (b) baseline patient characteristics; (c) clinical features and treatment regimens; (d) survival and toxicity data; and (e) evaluation indicators of quality and bias.

### Quality and bias assessments

The Joanna Briggs Institute criteria were used to assess the quality and bias of the included literature [14], which were independently completed by two researchers (QZ and MD) (Table 1) [15–25].

**Table 1:** Assessment of risk of bias in included studies

Study	Criterion									
	a	b	c	d	e	f	g	h	i	j
*Japan*										
Mizoe 2009 [15]	Yes	Yes	Yes	Yes	No	Yes	Yes	Yes	No	Yes
Imai et 2011 [16]	Yes	Yes	Yes	Yes	No	Yes	Yes	Yes	No	Yes
Imai et 2016 [17]	Yes	Yes	Yes	Yes	No	Yes	Yes	Yes	No	Yes
Koto 2020 [18]	Yes	Yes	Yes	Yes	No	Yes	Yes	Yes	No	Yes
Demizu 2021 [19]	Yes	Yes	Yes	Yes	No	Yes	Yes	Yes	No	Yes
Shiba et 2021 [20]	Yes	Yes	Yes	Yes	No	Yes	Yes	Yes	No	Yes
Aoki 2022 [21]	Yes	Yes	Yes	Yes	No	Yes	Yes	Yes	No	Yes
*Italy*										
Evangelisti 2019 [22]	Yes	Yes	Yes	Yes	No	Yes	Yes	Yes	No	Yes
Iannalfi 2020 [23]	Yes	Yes	Yes	Yes	No	Yes	Yes	Yes	No	Yes
*Germany*										
Uhl 2014 [24]	Unclear	Yes	Yes	Yes	No	Yes	Yes	Yes	No	Yes
Mattke 2023 [25]	Yes	Yes	Yes	Yes	No	Yes	Yes	Yes	No	Yes

(a) Were there clear criteria for inclusion in the case series?; (b) Was the condition measured in a standard, reliable way for all participants included in the case series?; (c) Were valid methods used for identification of the condition for all participants included in the case series?; (d) Did the case series have consecutive inclusion of participants?; (e) Did the case series have complete inclusion of participants?; (f) Was there clear reporting of the demographics of the participants in the study?; (g) Was there clear reporting of clinical information of the participants?; (h) Were the outcomes or follow-up results of cases clearly reported?; (i) Was there clear reporting of the presenting sites’/clinics’ demographic information?; (j) Was statistical analysis appropriate?

### Statistical analysis

Baseline variables and incidence of toxicity were analysed using descriptive statistics. Data descriptions included frequencies and percentages for dichotomous data, and means with standard deviations or medians with interquartile ranges for continuous data. We used a random effects model to provide an overall pooled estimate for the case series studies. We computed proportions with 95% confidence intervals (CIs) to estimate the effect sizes for continuous outcomes. All the analyses were performed using STATA version 14.0 (StataCorp, College Station, Texas, USA).

## Results

### Search strategy

**Fig. 1:**
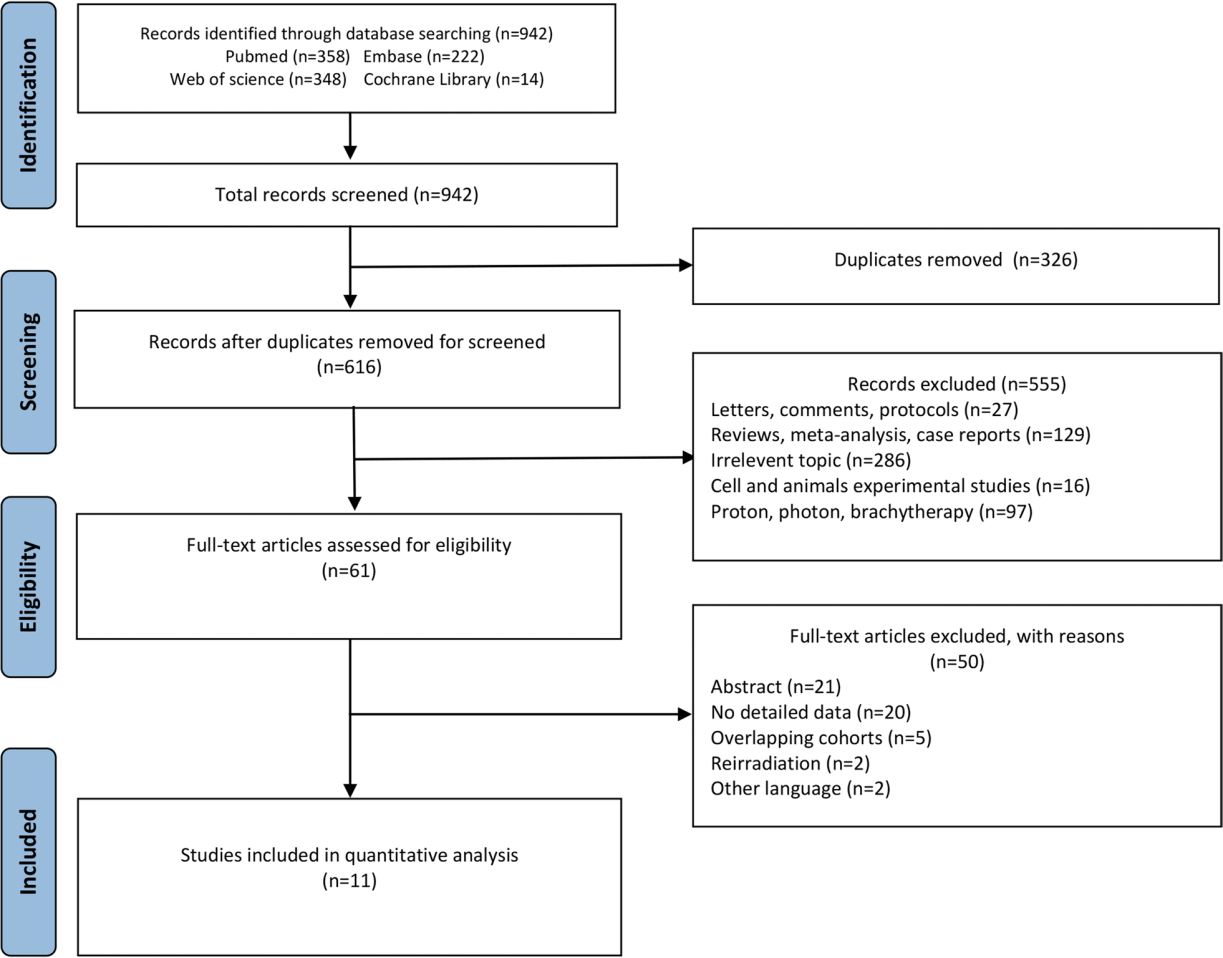
Search results per the PRISMA guidelines

A total of 942 candidate articles were identified through the systematic literature search (Fig. 1). Based on the exclusion criteria, 61 relevant studies were screened. As demonstrated in Fig. 1, we excluded another 50 additional items. Eleven studies from different regions were included as follows (Table 1): Japan (n = 7), Italy (n = 2), and Germany (n = 2). In terms of research design, more than half were retrospective studies (n = 7), and the other four were prospective studies (n = 3) and phase I/II or II trials (n = 1) (Table 2) [15–25].

**Table 2:** Baseline characteristics of included studies

Study	Institution	Study type	Outcomes	Period	No. of patients	Median age (year)	Male/female	Median follow-up (month)
Mizoe 2009 [15]	Japan (NIRS)	phase I/II or II	Survival, toxicity	1995–2007	33	47 (16–76)	14/19	53 (8–129)
Imai 2011 [16]	Japan (NIRS)	retrospective	Survival, toxicity	1996–2007	95	66 (30–85)	68/27	42 (13–112)
Imai 2016 [17]	Japan (NIRS)	retrospective	Survival, toxicity	1996–2013	188	66 (26–87)	128/60	62 (6.8–147.5)
Koto 2020 [18]	Japan (NIRS)	retrospective	Survival, toxicity	2002–2016	34	52 (16–76)	18/16	108 (9–175)
Demizu 2021 [19]	Japan (Multicentre)*	retrospective	Survival, toxicity	2003–2014	219	67 (26–87)	151/68	56 (7–132)
^§^Shiba 2021 [20]	Japan (GHMC)	prospective	Survival, toxicity	2011–2019	32	67 (27–84)	24/8	36.9 (4.4–96.4)
Aoki 2022 [21]	Japan (NIRS)	retrospective	Survival, toxicity	2005–2014	19	63 (26–81)	6/13	68 (29–144)
Evangelisti 2019 [22]	Italy (CNAO)	prospective	Survival, toxicity	2013–2016	18	64.7 (38–83)	12/6	23.3 (6–47)
^†^Iannalfi 2020 [23]	Italy (CNAO)	prospective	Survival, toxicity	2011–2018	65	58 (13–81)	42/23	49 (6–87)
Uhl 2014 [24]	Germany (HIT)	retrospective	Survival, toxicity	1998–2008	155	48(15–85)	76/79	72(12–165)
^†^Mattke 2023 [25]	Germany (HIT)	retrospective	Survival, toxicity	2009–2014	111	51	63/48	52.2

*HIBMC, QST, SAGA-HIMAT, GHMC

^†^Only carbon ion data is included

^§^Only chordoma data is included

*NIRS* National Institute of Radiological Sciences, *HIBMC* Hyogo Ion Beam Medical Center, *QST* National Institutes for Quantum Science and Technology, *SAGA-HIMAT* SAGA Heavy Ion Medical Accelerator in Tosu, *GHMC* Gunma University Heavy Ion Medical Center, *CNAO* Clinical Department, National Center for Oncological Hadrontherapy, *HIT* Heidelberg Ion-Beam Therapy Center

### Baseline characteristics

As listed in Table 2, C-ion RT was performed on 969 patients with chordomas from seven different research institutions. All 11 included studies reported primary endpoints (LC and OS) and secondary endpoints (toxicity) after C-ion RT. Table 2 summarizes the main details of the patients’ baseline characteristics in all the included studies [15–25].

### Clinical features and treatment regimens

In total, 969 patients were pathologically diagnosed with chordomas. Regarding lesion location, 57% (n = 552) of the tumours arose in the sacral region, 41 (n = 398) in the skull base, and 2 (n = 19) in the spine (upper cervical). Nine studies have reported on tumour status; the recurrent presentations were 13.2% (n = 119) and primary presentations were 86.8% (n = 785). Table 3 summarizes the main details of the tumour status, histology, surgery, and chemotherapy [15–25].

**Table 3:** Clinical features and treatment regimens main results of all included studies

Study	Histology	Tumor status P/R/M	Tumor site	Median target volume (cc)	Surgery	Chemotherapy	Beam-delivery	Total dose Gy (RBE)	Fractions (n)	Dose/fraction Gy (RBE)
Mizoe 2009 [15]	Chordoma	NR	Skull base	32 (2–328)	33 (100%)	0	Passive scanning	48.0–60.8	16	3.0–3.8
Imai 2011 [16]	Chordoma	84/11/0	Sacral	370 (47–1468)	11 (11.6%)	0	Passive scanning	70.4 (52.8–73.6)	16	3.3–4.6
Imai 2016 [17]	Chordoma	188/0/0	Sacral	345 (42–1497)	0	0	Passive scanning	67.2 (64–73.6)	16	4.0–4.6
Koto 2020 [18]	Chordoma	27/7/0	Skull base	18.7 (1.5–126.7)	29 (85.3%)	0	Passive scanning	60.8	16	3.8
Demizu 2021 [19]	Chordoma	219/0/0	Sacral	Unclear	8 (3.7%)	0	Passive scanning	67.2 (67.2–79.2)	16 (16–32)	4.2 (2.2–4.4)
^§^Shiba 2021 [20]	Chordoma	NR	Sacral	205.7 (1.6–2074.3)	5 (15.6$)	0	NR	67.2 (64–67.2)	16	4.0–4.2
Aoki 2022 [21]	Chordoma	17/2/0	Spine	39.3 (9.11–117.93)	0	0	NR	60.8	16	3.8
Evangelisti 2019 [22]	Chordoma	18/0/0	Sacral	374(51.6–1740)	0	0	Active scanning	70.4	16	4.4
^†^Iannalfi 2020 [23]	Chordoma	46/19/0	Skull base	13 (0.4–87.4)	61 (93.8%)	0	Active scanning	70.4	16	4.4
Uhl 2014 [24]	Chordoma	101/54/0	Skull base	70 (2–294)	139 (89.7%)	0	Active scanning	60	20	3.0
^†^Mattke 2023 [25]	Chordoma	85/26/0	Skull base	40.9	NR	0	Active scanning	66	22	3.0

*NR* no reported, *P/R/M* primary/recurrent/metastasis, *RBE* relative biological effectiveness

^†^Only carbon ion data is included

^§^Only chordoma data is included

### C-ion RT

In terms of C-ion RT, nine studies have reported on the beam delivery mode (Table 3). Radiation oncologists in Japan tend to use passive scanning, whereas those in Germany and Italy tend to use active scanning. Overall, the median target volume was 13 374 cc. Regarding dose regimens (Table 3), significant differences among different research institutions were observed [15–25].

### Pooled incidences of LC

**Fig. 2:**
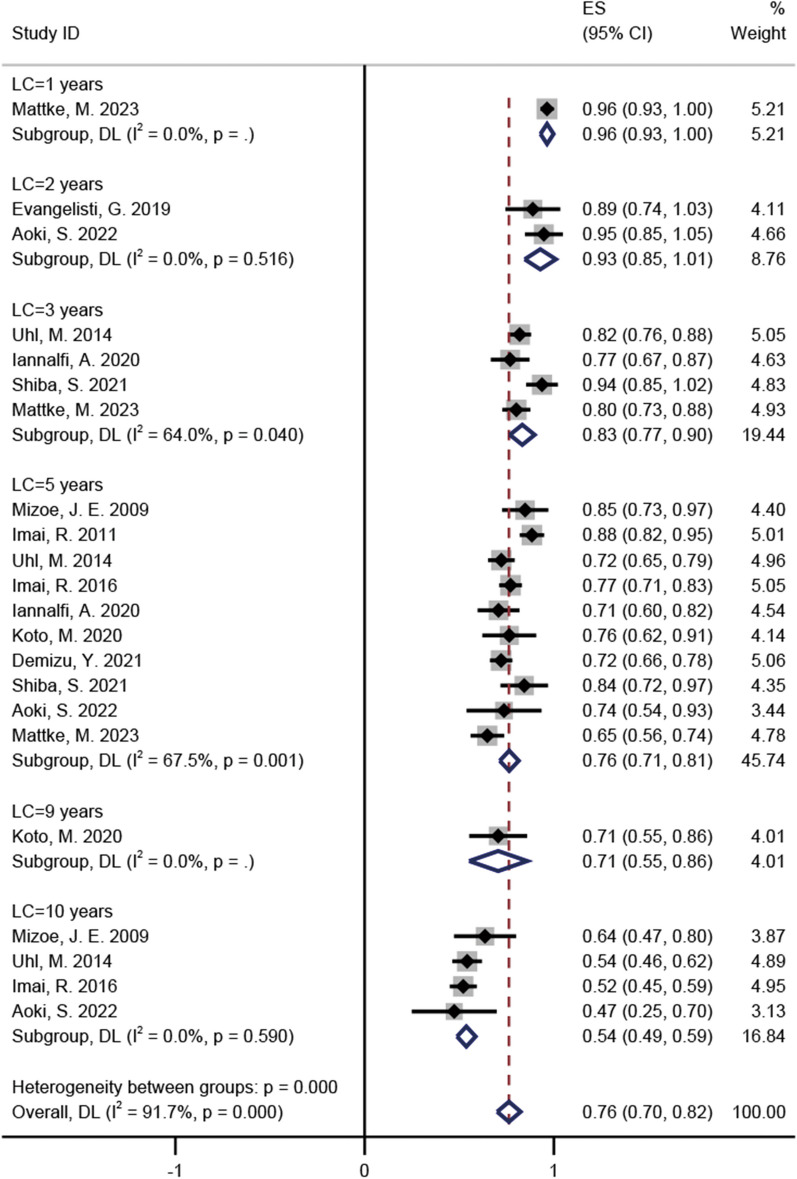
The pooled incidences of LC after C-ion RT for chordoma

The LC incidence at 1-, 2-, 3-, 5-, 9-, and 10-years in these studies were 96% (95% CI = 0.93–1, *I*^2^ = 0%), 93% (95% CI = 0.85–1.01, *I*^2^ = 0%), 83% (95% CI = 0.77–0.9, *I*^2^ = 64%), 76% (95% CI = 0.71–0.81, *I*^2^ = 67.5%), 71% (95% CI = 0.55–0.86, *I*^2^ = 0%), and 54% (95% CI = 0.49–0.59, *I*^2^ = 0%) respectively (Fig. 2) [15–25]. For the five studies regarding skull base chordomas, LC incidence at 1-, 3-, 5-, 9-, and 10-years were 96% (95% CI = 0.93–1, *I*^2^ = 0%), 80% (95% CI = 0.76–0.85, *I*^2^ = 0%), 73% (95% CI = 0.67–0.79, *I*^2^ = 43.9%), 71% (95% CI = 0.55–0.86, *I*^2^ = 0%), and 56% (95% CI = 0.49–0.63, *I*^2^ = 3.4%), respectively (Additional file 1: Fig. S1) [15, 18, 23–25]. For the five studies regarding sacral chordomas, LC incidence at 2-, 3-, 5-, and 10-years were 89% (95% CI = 0.74–1.03, *I*^2^ = 0%), 94% (95% CI = 0.85–1.02, *I*^2^ = 0%), 80% (95% CI = 0.72–0.88, *I*^2^ = 79.2%), and 52% (95% CI = 0.45–0.59, *I*^2^ = 0%), respectively (Additional file 2: Fig. S2) [16, 17, 19, 20, 22].

### Pooled incidences of OS

**Fig. 3:**
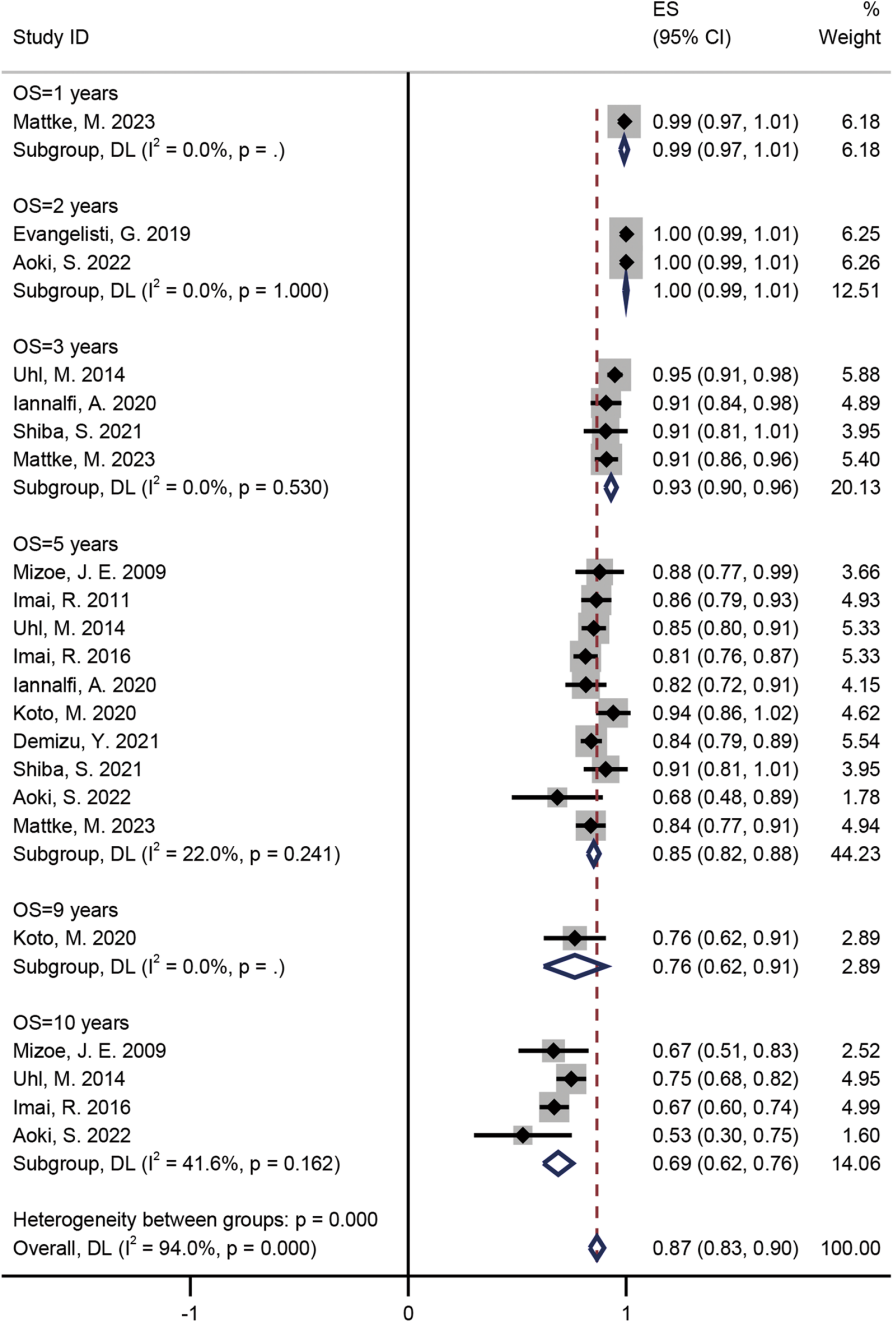
The pooled incidences of OS after C-ion RT for chordoma

As presented in Fig. 3, after undergoing CIRT for 1-, 2-, 3-, 5-, 9-, and 10-years, the OS rates for chordomas were 99% (95% CI = 0.97–1.01, *I*^2^ = 0%), 100% (95% CI = 0.99–1.01, *I*^2^ = 0%), 93% (95% CI = 0.9–0.96, *I*^2^ = 0%), 85% (95% CI = 0.82–0.88, *I*^2^ = 22%), 76% (95% CI = 0.62–0.91, *I*^2^ = 0%), and 69% (95% CI = 0.62–0.76, *I*^2^ = 41.6%), respectively (Fig. 3) [15–25]. For different tumour sites, the OS rates at 1-, 3-, 5-, 9-, and 10-years for skull base chordomas were 99% (95% CI = 0.97–1.01, *I*^2^ = 0%), 93% (95% CI = 0.91–0.96, *I*^2^ = 0%), 86% (95% CI = 0.82–0.90, *I*^2^ = 27.1%), 76% (95% CI = 0.62–0.91, *I*^2^ = 0%), and 74% (95% CI = 0.67–0.80, *I*^2^ = 0%), respectively (Additional file 3: Fig. S3) [15, 18, 23–25]; the OS rates at 2-, 3-, 5-, and 10-years for sacral chordomas were 100% (95% CI = 0.99–1.01, *I*^2^ = 0%), 91% (95% CI = 0.81–1.01, *I*^2^ = 0%), 84% (95% CI = 0.81–0.87, *I*^2^ = 0%), and 67% (95% CI = 0.60–0.74, *I*^2^ = 0%), respectively (Additional file 4: Fig. S4) [16, 17, 19, 20, 22].

### Toxicity

Assessment on the toxicity of C-ion RT is presented in Table 4 [15–25]. Our systematic review observed grade 3 and 4 acute toxicity, with incidences of 2.9–5.3% and 0.5%, respectively [16, 18, 19, 21]. For grade 4 and 5 late toxicities, the incidences were 2.1–5.9% and 2.9%, respectively [16–19]. A study on skull base chordomas identified grade 3 acute and grade 4 and 5 late toxicities; the incidence were 2.9% and 2.9–5.9%, respectively [18]. Serious late toxicities included mucositis (grade 5, n = 1) and optic nerve injury (grade 4, n = 2) [18]. Regarding chordomas in the sacral region, grade 4 acute and late toxicities were observed, with incidences of 0.5% and 1.1–2.3%, respectively [16, 17, 19]. The major radiation-induced acute and late toxicities were skin erythema or dermatitis, gastrointestinal tract, neuropathy, myositis, insufficiency fracture, pain, and urinary retention. Serious late toxicities included skin erythema or dermatitis (grade 4, n = 8) and pain (grade 4, n = 1) [16, 17, 19]. In one study on chordomas in the spine (upper cervical), toxicity above grade 3 was not observed [16]. The late toxicities included dysphagia (n = 1), myelitis (n = 1), encephalomyelitis (n = 5), and vertebral compression fractures (n = 5) [23].

**Table 4:** Survival outcomes, toxicity incidence and prognostic factors on patients of all included studies

Study	Local recurrence	Metastasis	Local control	Overall survival	Toxicity	Prognostic factors been evaluated
Mizoe 2009 [15]	Unclear	NR	5-y (85.1%) 10-y (63.8%)	5-y (87.7%) 10-y (67.0%)	Acute: ≤ G2 Late: ≤ G2 (G2 = 3%)	Age, Sex, KPS, Dose, GTV volume
Imai 2011 [16]	6 (6.3%)	NR	5-y (88.0%)	5-y (86.0%)	Acute: ≤ G3 (G3 = 3.2%) Late: ≤ G4 (G3 = 2.1%, G4 = 2.1%)	NR
Imai 2016 [17]	41 (21.8%)	54 (28.7%)	5-y (77.2%) 10-y (52.0%)	5-y (81.1%) 10-y (66.8%)	Acute: NR Late: ≤ G4 (G4 = 1.1%)	Sex, Tumor volume (≤ 500 cc or > 500 cc), Level of proximal invasion (≥ S2 or < S2), Total irradiated dose (≤ 67.2GyE or > 67.2GyE)
Koto 2020 [18]	11 (32.4%)	3 (8.8%)	5-y (76.9%) 9-y (69.2%)	5-y (93.5%) 9-y (77.4%)	Acute: ≤ G3 (G2 = 20.6%, G3 = 2.9%) Late: ≤ G5 (G3 = 2.9%, G4 = 5.9%, G5 = 2.9%)	Sex, Age(> 52 or ≤ 52), Performance status(0/1/2), **Tumor status (Naïve/Recurrence/Residua tumor)**^**b**^, Eye symptom(yes or no), **GTV volume (> 34.7 cc or ≤ 34.7 cc)**^**a,b**^, **D1cc (> 58.9GyE or ≤ 58.9GyE)**^**b**^
Demizu 2021 [19]	61 (27.9%)	39 (17.8%)	5-y (72.0%)	5-y (84.0%)	Acute: ≤ G4 (G3 = 3.2%, G4 = 0.5%) Late: ≤ G4 (G3 = 3.7%, G4 = 2.3%)	**Age (≥ 67 or < 67)**^**b, c**^, Sex, Performance status, **PTV volume (≥ 500 cc or < 500 cc)**^**b**^
^§^Shiba 2021 [20]	8 (15.1%)	11 (20.8%)	3-y (92.5%) 5-y (84.8%)	3-y (91.3%) 5-y (91.3%)	Acute: ≤ G2 Late: ≤ G3	Age, Sex, Chemotherapy, **Performance status (0–1 or 2–3)**^**c**^, Prior treatment, Distance of tumor-GI (≤ 3 mm or > 3 mm), Distance of tumor-GI (≤ 5 mm or > 5 mm), **GTV volume (≤ 300 cc or > 300 cc)**^**a**^, GTV D_98_ (≤ 64GyE or > 64GyE), GTV D_95_ (≤ 66GyE or > 66GyE), GTV V_64_ (≤ 98 or > 98), GTV V_60_ (≤ 98 or > 98), GTV V_<64_ (≤ 1cm^3^ or > 1cm^3^), **GTV V**_**<60**_** (≤ 1cm**^**3**^** or > 1cm**^**3**^**)**^**a**^
Aoki 2022 [21]	7 (36.8%)	5 (26.3%)	2-y (94.7%) 5-y (75.2%) 10-y (46.4%)	2-y (100%) 5-y (68.4%) 10-y (52.1%)	Acute: ≤ G3 (G3 = 5.3%) Late: ≤ G3 (G3 = 10.5%)	Age, Sex, KPS, Tumor status (initial or recurrent), **GTV volume (> 40 cc or ≤ 40 cc)**^**b**^, Spinal cord infiltration, Minimum dose of GTV
Evangelisti 2019 [22]	2 (11.%)	0	2-y (89.0%)	2-y (100%)	Acute: ≤ G1 (G1 = 27.8%) Late: ≤ G3 (G2 or G3 = 16.7%)	NR
^†^Iannalfi 2020 [23]	14 (21.5%)	9 (13.8%)	3-y (77.0%) 5-y (71.0%)	3-y (90.0%) 5-y (82.0%)	Acute: ≤ G2 Late: Unclear	Sex, Age, Histology, Anatomic extension of the disease (upper/middle/lower clivus), **GTV volume (≤ 23.1 cc or > 23.1 cc)**^**a,b**^, **Optic pathways and brainstem compression**^**a**^, Target coverage (D95% of CTV-HR and GTV)
Uhl 2014 [24]	55 (35.5%)	4 (2.6%)	3-y (82.0%) 5-y (72.0%) 10-y (54.0%)	3-y (95.0%) 5-y (85.0%) 10-y (75.0%)	Acute: NR Late: Quantitative toxicity results	**PTV volume (< 100 ml or ≥ 100 ml)**^**a**^**, Total dose (≤ 51 GyE or > 51 GyE)**^**a**^
^†^Mattke 2023 [25]	NR	NR	1-y (96.1%) 3-y (80.4%) 5-y (64.5%)	1-y (99.0%) 3-y (91.2%) 5-y (83.3%)	Acute: ≤ G1 Late: ≤ G3	Age, Sex, Tumor status (primary or recurrent)

Bold indicates statistically significant difference

*NR* no reported, *KPS* Karnofsky performance status, *GTV* gross target volume, *PTV* plan target volume, *GI*: gastrointestinal

^†^Only carbon ion data is included

^§^Only chordoma data is included

^**a**^Factor significantly correlated with local control (LC) (p ≤ 0.05)

^**b**^Factor significantly correlated with overall survival (OS) (p ≤ 0.05)

^**c**^Factor significantly correlated with progress-free survival (PFS) (p ≤ 0.05)

### Prognostic factors of C-ion RT effectiveness

The following factors were evaluated in nine studies: age, sex, Karnofsky performance status, gross target volume, planning target volume, tumour volume, level of proximal invasion, total irradiated dose, tumour status, eye symptoms, prior treatment, tumour-gastrointestinal distance, chemotherapy, spinal cord infiltration, and histology. Table 4 (Boldface indicates statistically significant difference) shows the main details of the prognostic factors of C-ion RT effectiveness in all the included studies [15, 17–21, 23–25].

## Discussion

Chordomas often occur adjacent to critical neuroaxes, such as the brainstem, spinal cord, and optic pathways [8, 9]. Therefore, complete resection of some chordomas is often difficult. A previous study has reported that the total resection rate of chordomas is approximately 20–70%, with an LC rate of 60–80% [26–29]. However, the LC rate in patients with subtotal resection is approximately 25–50% [26–29]. According to the practical guide from the Spine Oncology Society, RT plays an important role, especially PBT and C-ion RT, for some chordomas [30]. To the best of our knowledge, this is the first systematic review and meta-analysis of C-ion RT for chordomas, including the skull base, sacral, and mobile spine.

Achieving high doses of tumour irradiation is difficult with traditional photon therapy because of chordomas adjacent to the critical neuroaxis OAR. Performing photon irradiation therapy in patients postoperatively has also been reported. After five years, LC reached only 39% [31]. Satisfactory LC may be equally difficult to achieve with stereotactic body radiotherapy (SBRT) or stereotactic radiosurgery (SRS). Debus et al. have reported the results of SBRT in the treatment of chordomas, with a 5-year LC rate of only 50% [32]. Similarly, in a study of 93 patients with intracranial chordomas treated with SRS, the LC rate was 54.7% after 5 years [33]. However, C-ion RT demonstrated an impressive LC rate compared to photon therapy in our study, with pooled LC rates of 76% and 54% at 5 years and 10 years, respectively (Fig. 2).

C-ion RT and PBT have similar physical advantages (Bragg peaks). Moreover, the C ion has a better RBE than PBT. However, whether C-ion RT or PBT is more favourable for chordoma treatment remains a long-term challenge in the field of charged-particle therapy. A systematic review of RT for chordoma included 2 prospective and 21 retrospective studies of PBT published by Redmond et al. [30]. The median LC and OS after 5 years among the reported studies were 73.3% and 81.3%, respectively [30]. In our meta-analysis, the pooled incidence of LC and OS at 5 years were 76% and 85%, respectively (Figs. 2 and 3). Unfortunately, survival outcomes at follow-up times of ≥ 10 years could not be compared in the two studies. Using only prospective data, Iannalfi et al. have reported on PBT and C-ion RT for skull base chordomas [23]. They observed a 5-year LC rate of 84% in the PBT group and 71% in the C-ion RT group, although they stated that patients with poor prognosis were specifically assigned to the C-ion RT group [23]. An imbalance in the baseline of patients was observed, and comparing survival outcomes between the two techniques was not appropriate. The Heidelberg Ion Therapy Center has published a retrospective study of PBT and C-ion RT for skull base chordomas [25]. The 1-, 3-, and 5-year LC rates of the PBT group were 97%, 80%, and 61%, respectively, whereas those in the C-ion group were 96%, 80%, and 65%, respectively [25]. The corresponding 1-, 3-, and 5-year OS rates were 100%, 92%, and 92% for the PBT group and 99%, 91%, and 83% for the C-ion RT group [25]. Outcomes of C-ion RT and PBT treatment of skull base chordomas may be similar in terms of tumour control, survival, and toxicity [25]. Therefore, the advantages of different charged particle RT techniques for chordomas need to be determined in more prospective studies, especially randomised controlled clinical trials.

Of the 11 included articles, five were on skull base chordomas, five were on sacral chordomas, and one was regarding a mobile spine chordoma (Table 2). As reported in Additional files 1–4, the pooled LC and OS rates at 5 years and 10 years were very similar for the skull base and sacral chordomas, which indicates that C-ion RT may have similar tumour control and survival in different sites of chordomas [15–25]. Chordomas have various pathological subtypes (classic, chondroid, and dedifferentiated subtypes), among which the prognosis of the chondroid type and dedifferentiated subtypes is very different. A model of individualised C-ion RT for chordomas requires further investigation. To the best of our knowledge, no reports on particle therapy (C-ion or PBT) for the different pathological subtypes of chordoma have been published.

Regarding toxicity, the most frequent toxicity was skin reaction [15–17, 19, 20, 22], and the incidences of grade 3 acute and grade 4 late skin toxicity were 3.2% and 1.1–2.1%, respectively [16, 17, 19]. Kamada et al. have suggested that 73.6 Gy (RBE) may be the maximum tolerated dose for patients without subcutaneous tumours in order to reduce the incidence of skin toxicity [34]. For patients with subcutaneous tumour involvement, the maximum dose may not exceed 70.4 Gy (RBE) [34]. Koto et al. have reported on a one case with a recurrent skull base chordoma that developed grade 5 late toxicity (fatal bleeding from the nasopharynx) at 9 years and 3 months after C-ion RT [18]. This patient had undergone transsphenoidal approach surgery 3 years before C-ion RT, and received a second surgery through the transpetrosal approach 1 year prior to C-ion RT [18]. Late neuropathy (grade 3) and optic nerve injury toxicity (grade 4) had an incidence of 0.9–3.2% and 5.9%, respectively [17–19]. According to a review on RT for chordomas published by Redmond et al., the median follow-up time for photon RT was 10–50 months [30]. However, the median follow-up time in our included studies was longer, ranging from 23.3 to 108 months (Table 2). Therefore, directly comparing the toxicity between photon RT and C-ion RT is difficult.

This meta-analysis has several limitations. First, only 11 of 61 relevant full-text articles met the inclusion criteria, and grey literature was ignored, which may have a higher risk of publication bias. Second, the metadata were from different regions: Japan (64%), Italy (18%), and Germany (18%); only four studies were prospective or phase I/II or II trials. Therefore, the metadata were mainly obtained from small retrospective studies, and there could be patients lost to follow-up, selection biases, and reporting biases. Finally, chordomas are generally slow-growing; Scampa et al. have reported a median OS of approximately 10 years [35], although four studies had a follow-up period of > 10 years in our study.

Further, C-ion RT has some limitations as a therapeutic strategy for chordomas. Chordomas have various pathological subtypes (classic, chondroid, and dedifferentiated subtypes), among which the chondroid subtype has the best prognosis, and the dedifferentiated subtype has the worst prognosis [36–38]. Therefore, it is interesting to hypothesise whether the dose is sufficient for pathological types with poor prognosis. In addition, clinical observations have demonstrated the metastatic potential of chordomas, with 5–40% of patients developing distant metastases during the course of the disease [39]. Moreover, systemic therapies, including chemotherapy and targeted therapy, may be occasionally necessary, although no recommended consensus or treatment guidelines have been established.

## Conclusion

C-ion RT has attractive clinical application prospects and is an important local treatment strategy for chordomas. Encouraging results were observed in terms of LC and OS. Meanwhile, the acute and late toxicities were acceptable.

### Supplementary Information

**Additional file 1: Fig. S1**. The pooled incidences of LC after C-ion RT for skull base chordoma

**Additional file 2: Fig. S2**. The pooled incidences of OS after C-ion RT for skull base chordoma

**Additional file 3: Fig. S3**. The pooled incidences of LC after C-ion RT for sacral chordoma

**Additional file 4: Fig. S4**. The pooled incidences of OS after C-ion RT for sacral chordoma

## Data Availability

Data and material are available on request.

## Abbreviations

C-ion RT: Carbon ion radiotherapy
LC: Local control
CI: Confidence interval
OS: Overall survival
RT: Radiotherapy
OAR: Organs at risk
PBT: Proton beam therapy
CI(s): Confidence interval(s)
RBE: Relative biological effectiveness
SBRT: Stereotactic body radiotherapy
SRS: Stereotactic radiosurgery

## References

[1] Salisbury JR, Deverell MH, Cookson MJ (1993). Three-dimensional reconstruction of human embryonic notochords: clue to the pathogenesis of chordoma. J Pathol.

[2] Smoll NR, Gautschi OP, Radovanovic I (2013). Incidence and relative survival of chordomas: the standardized mortality ratio and the impact of chordomas on a population. Cancer.

[3] Flanagan AM, Yamaguchi T, Fletcher CDM (2013). Chordoma. World Health Organization (WHO) classification of tumours of soft tissue and bone.

[4] Bjornsson J, Wold LE, Ebersold MJ (1993). Chordoma of the mobile spine. A clinicopathologic analysis of 40 patients. Cancer.

[5] Pan Y, Lu L, Chen J (2018). Analysis of prognostic factors for survival in patients with primary spinal chordoma using the SEER Registry from 1973 to 2014. J Orthop Surg Res.

[6] Zuckerman SL, Bilsky MH, Laufer I (2018). Chordomas of the skull base, mobile spine, and sacrum: an epidemiologic investigation of presentation, treatment, and survival. World Neurosurg.

[7] Noël G, Feuvret L, Ferrand R (2004). Radiotherapeutic factors in the management of cervical-basal chordomas and chondrosarcomas. Neurosurgery.

[8] Hug EB, Loredo LN, Slater JD (1999). Proton radiation therapy for chordomas and chondrosarcomas of the skull base. J Neurosurg.

[9] Samii A, Gerganov VM, Herold C (2007). Chordomas of the skull base: surgical management and outcome. J Neurosurg.

[10] Pennicooke B, Laufer I, Sahgal A (2016). Safety and local control of radiation therapy for chordoma of the spine and sacrum: a systematic review. Spine.

[11] Pearlman AW, Friedman M (1970). Radical radiation therapy of chordoma. Am J Roentgenol Radium Ther Nucl Med.

[12] Hamada N, Imaoka T, Masunaga S (2010). Recent advances in the biology of heavy-ion cancer therapy. J Radiat Res.

[13] Moher D, Liberati A, Tetzlaff J (2009). Preferred reporting items for systematic reviews and meta-analyses: the PRISMA statement. PLoS Med.

[14] Munn Z, Barker TH, Moola S (2020). Methodological quality of case series studies: an introduction to the JBI critical appraisal tool. JBI Evid Synth.

[15] Mizoe JE, Hasegawa A, Takagi R (2009). Carbon ion radiotherapy for skull base chordoma. Skull Base.

[16] Imai R, Kamada T, Sugahara S (2011). Carbon ion radiotherapy for sacral chordoma. Br J Radiol.

[17] Imai R, Kamada T, Araki N (2016). Carbon ion radiation therapy for unresectable sacral chordoma: an analysis of 188 cases. Int J Radiat Oncol Biol Phys.

[18] Koto M, Ikawa H, Kaneko T (2020). Long-term outcomes of skull base chordoma treated with high-dose carbon-ion radiotherapy. Head Neck.

[19] Demizu Y, Imai R, Kiyohara H (2021). Carbon ion radiotherapy for sacral chordoma: a retrospective nationwide multicentre study in Japan. Radiother Oncol.

[20] Shiba S, Okamoto M, Kiyohara H (2021). Impact of carbon ion radiotherapy on inoperable bone sarcoma. Cancers (Basel).

[21] Aoki S, Koto M, Ikawa H (2022). Long-term outcomes of high dose carbon-ion radiation therapy for unresectable upper cervical (C1–2) chordoma. Head Neck.

[22] Evangelisti G, Fiore MR, Bandiera S (2019). Carbon ions therapy as single treatment in chordoma of the sacrum Histologic and metabolic outcome studies. Eur Rev Med Pharmacol Sci.

[23] Iannalfi A, D'Ippolito E, Riva G (2020). Proton and carbon ion radiotherapy in skull base chordomas: a prospective study based on a dual particle and a patient-customized treatment strategy. Neuro Oncol.

[24] Uhl M, Mattke M, Welzel T (2014). Highly effective treatment of skull base chordoma with carbon ion irradiation using a raster scan technique in 155 patients: first long-term results. Cancer.

[25] Mattke M, Ohlinger M, Bougatf N (2023). Proton and carbon ion beam treatment with active raster scanning method in 147 patients with skull base chordoma at the Heidelberg Ion Beam Therapy Center-a single-center experience. Strahlenther Onkol.

[26] Ozaki T, Hillmann A, Winkelmann W (1997). Surgical treatment of sacrococcygeal chordoma. J Surg Oncol.

[27] Yonemoto T, Tatezaki S, Takenouchi T (1999). The surgical management of sacrococcygeal chordoma. Cancer.

[28] Cheng EY, Ozerdemoglu RA, Transfeldt EE (1999). Lumbosacral chordoma. Prognostic factors and treatment. Spine.

[29] Bergh P, Kindblom LG, Gunterberg B (2000). Prognostic factors in chordoma of the sacrum and mobile spine: a study of 39 patients. Cancer.

[30] Redmond KJ, Schaub SK, Lo SL (2023). Radiotherapy for mobile spine and sacral chordoma: a critical review and practical guide from the spine tumor academy. Cancers (Basel).

[31] Forsyth PA, Cascino TL, Shaw EG (1993). Intracranial chordomas: a clinicopathological and prognostic study of 51 cases. J Neurosurg.

[32] Debus J, Schulz-Ertner D, Schad L (2000). Stereotactic fractionated radiotherapy for chordomas and chondrosarcomas of the skull base. Int J Radiat Oncol Biol Phys.

[33] Pikis S, Mantziaris G, Peker S (2022). Stereotactic radiosurgery for intracranial chordomas: an international multiinstitutional study. J Neurosurg.

[34] Kamada T, Tsujii H, Tsuji H (2002). Efficacy and safety of carbon ion radiotherapy in bone and soft tissue sarcomas. J Clin Oncol.

[35] Scampa M, Tessitore E, Dominguez DE (2022). Sacral chordoma: a population-based analysis of epidemiology and survival outcomes. Anticancer Res.

[36] Barnes L, Kapadia SB (1994). The biology and pathology of selected skull base tumors. J Neurooncol.

[37] Mitchell A, Scheithauer BW, Unni KK (1993). Chordoma and chondroid neoplasms of the spheno-occiput. An immunohistochemical study of 41 cases with prognostic and nosologic implications. Cancer.

[38] Jian BJ, Bloch OG, Yang I (2010). Adjuvant radiation therapy and chondroid chordoma subtype are associated with a lower tumor recurrence rate of cranial chordoma. J Neurooncol.

[39] McPherson CM, Suki D, McCutcheon IE (2006). Metastatic disease from spinal chordoma: a 10-year experience. J Neurosurg Spine.

